# The Current State of the Art in PAMAM and PLL Dendrimers, Boron Clusters, and Their Complexes for Biomedical Use

**DOI:** 10.3390/biomedicines14030615

**Published:** 2026-03-10

**Authors:** Agnieszka Maria Kołodziejczyk, Edyta Błaszczyk, Bolesław T. Karwowski

**Affiliations:** Nucleic Acids Damage Laboratory of Food Science Department, Faculty of Pharmacy, Medical University of Lodz, ul. Muszyńskiego 1, 90-151 Lodz, Poland; edyta.blaszczyk@umed.lodz.pl

**Keywords:** boron neutron capture therapy, PAMAM and PLL dendrimers, dendrimer–boron clusters

## Abstract

Poly(amidoamine) (PAMAM) and poly-*L*-lysine (PLL) dendrimers have emerged as highly versatile macromolecular platforms with significant potential in biomedical applications, owing to their well-defined architecture, tunable surface chemistry, and capacity for multivalent functionalization. Their ability to carry substantial molecular payloads and to be engineered for selective interactions with biological systems has positioned them as attractive candidates for targeted drug delivery, including the transport of boron-rich compounds. Recent advances in dendrimer chemistry have enabled the incorporation of boron clusters into PAMAM and PLL structures, creating hybrid systems designed to enhance cellular uptake, improve tumor selectivity, and increase boron accumulation within malignant tissues. Given the growing interest in boron neutron capture therapy (BNCT), the integration of boron clusters into dendrimer structures represents a particularly promising direction for enhancing boron delivery to tumors. This manuscript reviews current knowledge on PAMAM and PLL dendrimers and their boron-functionalized derivatives, summarizing findings from cell culture studies, in vivo models, and clinical or preclinical investigations. Particular attention is given to both the advantageous properties of these dendrimers—such as improved delivery efficiency and biocompatibility—and their potential undesirable biological effects. As such, PAMAM and PLL dendrimers represent an important and evolving class of carriers that may significantly advance the effectiveness of boron neutron capture therapy (BNCT) in cancer treatment.

## 1. Introduction

Dendrimers are nanometer-sized, extensively branched polymers that possess distinctive physicochemical characteristics—such as high surface charge density, uniform molecular structure, defined dimensions, and a tunable number of terminal groups—which make them highly suitable for biomedical applications. PAMAM and PLL dendrimers have been investigated for several decades with regard to their potential applications in medicine and pharmacy. Numerous reports describe their commercial use as carriers for drugs, vaccines, and genetic material, as well as in the design of diagnostic assays. PAMAM and PLL dendrimers exhibit inherent antifungal, antibacterial, and antimicrobial properties [1,2]. Despite the fact that PAMAM dendrimers also exhibit high cytotoxicity—dependent on their generation, size, surface charge and functional groups—numerous strategies for modifying the functional groups of PAMAM dendrimers have been described in the literature [3], enabling the reduction in this toxicity and thereby facilitating their broader application in biomedicine. Numerous functionalization strategies have also been developed for PLL dendrimers, enabling the reduction in their cytotoxicity and the targeted delivery to specific cell populations [4]. The use of dendrimers as drug carriers may enhance drug bioavailability and reduce the number of required doses. Another noteworthy aspect of PAMAM and PLL dendrimer applications is the formation of conjugates with borane cages. Owing to their high boron content, such systems can be effectively tested and employed in Boron Neutron Capture Therapy (BNCT) for cancer treatment [5]. Boron cages are characterized by an ellipsoidal or spherical geometry and a three-dimensional atomic arrangement, which facilitates the design of complex spatial molecular architectures [6]. They also exhibit unique capacities for forming non-covalent interactions, distinguishing them from classical organic molecules due to their specific modes of interaction with biological targets. These structures display high chemical stability while remaining amenable to functionalization [7]. Notably, they are resistant to ionizing radiation [8], a property that is crucial in the context of radiopharmaceutical design. Borane cages, including boranes and carboranes, can also be utilized as drug-delivery systems, antimicrobial agents effective against bacteria, fungi, and viruses, as well as in molecular imaging [9].

**Figure 1:**
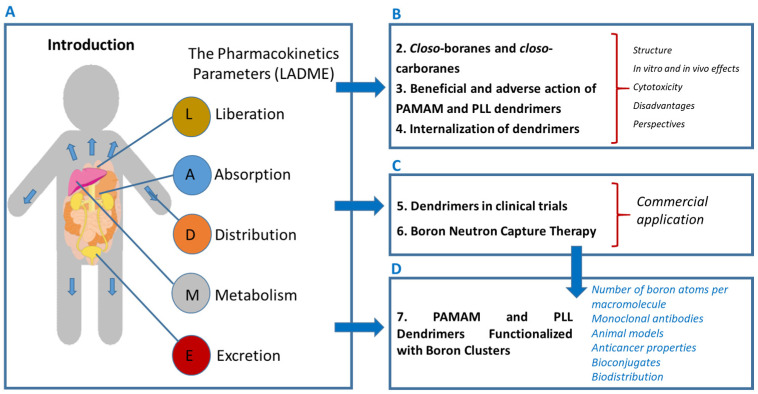
The schematically illustrated sections of the manuscript including the Introduction (**A**), boron clusters and PAMAM and PLL dendrimers and their internalization (**B**), dendrimer application in clinical trials and boron capture therapy (**C**) and dendrimers functionalized with boron clusters with particular emphasis on their anticancer properties (**D**).

In drug-delivery systems, both the active pharmaceutical ingredient and the applied nanocarrier undergo the classical pharmacokinetic processes of liberation, absorption, distribution, metabolism, and excretion (LADME) [10,11]. In the development of new therapeutics, establishing rational design principles is of fundamental importance, as it enables the minimization of drug–drug interaction risks and a reduction in pharmacological losses [12]. An equally critical component is the detailed characterization of physicochemical properties that determine the course of LADME processes [11]. With the exception of oligonucleotides used in gene therapy, every active pharmaceutical ingredient must penetrate the intracellular or intercellular space, elicit the desired biological effect, and subsequently undergo metabolism and/or be eliminated from the body [13]. Another important consideration is the route of drug administration, which may be either oral or parenteral [14]. Oral administration is non-invasive; however, it is affected by numerous factors, including the solubility of the active pharmaceutical ingredient, the permeability of the mucosal membrane, and its stability in the gastrointestinal environment [15,16]. In contrast, parenteral administration encompasses issues such as the types of parenteral formulations (solutions, suspensions, emulsions, and implants), the role of excipients, and technological capabilities enabling drug delivery through systems such as liposomes, nanoparticles, and sustained-release formulations [17,18]. Figure 1A presents a schematic overview of the LADME processes along with the corresponding sections of the manuscript (B, C, D). The relevance of LADME is critical for dendrimers, boron clusters, and dendritic structures functionalized with boron clusters alike. In this work, we highlight the biomedical significance of dendrimers, boron clusters, and dendritic structures functionalized with boron clusters. In the case of these nanostructures and conjugates, the relevance of LADME is critical.

The challenges associated with the development of boron-based nanocomposites and dendrimers encompass synthesis, structural characterization, determination of physicochemical parameters, and evaluation of cytotoxic activity at in vitro, ex vivo, and in vivo levels. These aspects are addressed in Section 2, *Closo-Boranes and Closo-Carboranes—Structure and Property Relations*, and Section 3, *Beneficial and Adverse Action of PAMAM and PLL Dendrimers*. Although this topic has been extensively studied, the interest in PAMAM dendrimers for applications in medical sciences—particularly in drug delivery, genetic material transport, and viral delivery—continues to expand. Additionally, the pathways of dendrimer internalization are described and visualized in Section 4. Section 5, *Dendrimers in Clinical Trials* outlines selected examples of the commercial use of these dendrimers as well as ongoing clinical trials. The next section, Section 6, *Boron Neutron Capture Therapy*, highlights both the advantages and the requirements of selective tumor targeting and the delivery of therapeutic boron concentrations [19] (Figure 1C). Boron neutron capture therapy is considered one of the more advantageous cancer treatment modalities compared with other forms of radiotherapy [19]. One of the main aims of this manuscript is to discuss the current state of knowledge on poly(amidoamine) (PAMAM) and poly-*L*-lysine (PLL) dendrimers functionalized with boron cage structures (Figure 1D) in medical applications. The current state of the art of this dendritic structures decorated with boron clusters is outlined in Section 7. This summary of the advantages and limitations of PAMAM- and PLL-based boron carriers opens discussion for their potential use in optimizing effective and personalized BNCT for oncology patients.

## 2. Closo-Boranes and Closo-Carboranes—Structure and Property Relations

*Closo*-boranes are inorganic compounds composed primarily of boron and hydrogen atoms that form closed, polyhedral cluster structures. Although they are inorganic compounds, *closo*-boranes have properties that make them interesting for biomedical research. Their structures can be modified to create suitable compounds for drug development and other therapeutic applications. Their unique architecture arises from the electronic nature of boron, which, due to its electron deficiency, is capable of forming three-center two-electron (3c–2e) bonds. Extensive electron delocalization across the entire cluster framework gives rise to three-dimensional (3D) aromaticity, which in turn imparts exceptional chemical, thermal, and electrochemical stability [20]. This stability ensures that the compounds remain intact in the body, allowing them to reach their target cells and retain their properties. The most stable boron clusters are represented by the dianions decahydro-*closo*-decaborate [B_10_H_10_]^2−^ and dodecahydro-*closo*-dodecaborate [B_12_H_12_]^2−^. In combination with alkali metal cations (Li^+^, Na^+^, K^+^), they form highly water-soluble salts that behave as strong electrolytes [21]. Furthermore, the toxicity of their sodium salts is described as low and comparable to that of sodium chloride, primarily due to sodium toxicity. High sodium ions concentrations increase osmolarity, causing electrolyte disturbances [21]. In the case of [B_12_H_12_]^2−^, it has been observed that this anion can interact with lipid membranes [22].

**Figure 2:**
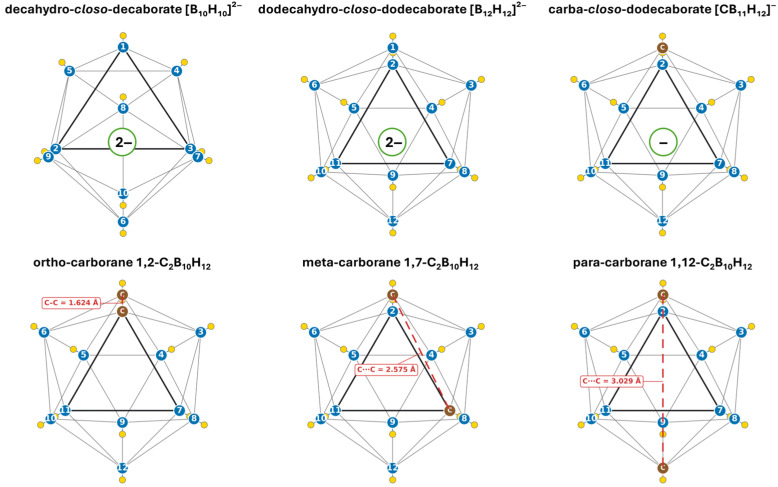
Schlegel-type projections depict selected *closo*-boranes and *closo*-carboranes including ortho-/meta-/para-C_2_B_10_H_12_, [B_10_H_10_]^2^^−^, [B_12_H_12_]^2^^−^, and [CB_11_H_12_]^−^ in a unified visual code (C = brown; B = blue with vertex numbering; H = yellow; cage edges = gray; projection face = thick black). For the carborane isomers, the annotated C···C separations are also provided in red with the values as follows: 1.624 Å (ortho, C–C), 2.575 Å (meta), and 3.029 Å (para).

The replacement of one or two boron atoms with carbon atoms results in the formation of *closo*-carboranes, including the monoanions carba-*closo*-carbadecaborate [CB_9_H_10_]^−^ and carba-*closo*-dodecaborate [CB_11_H_12_]^−^ and the neutral dicarba-*closo*-dodecaborate C_2_B_10_H_12_. The incorporation of carbon atoms alters the charge of clusters and markedly increases their hydrophobicity, which has a significant impact on their reactivity and chemical functionalization possibilities [23]. In the case of C_2_B_10_H_12_, it should be noted that this compound occurs in the form of three isomers, differing in the position of two carbon atoms in the cluster, which are 1,2-C_2_B_10_H_12_ (ortho-carborane), 1,7-C_2_B_10_H_12_ (meta-carborane), and 1,12-C_2_B_10_H_12_ (para-carborane) [20,23]. The placement of the carbon atoms within the cluster directly influences its hydrophilicity and polarity, which follow the order: ortho > meta > para. Furthermore, the ortho-isomer is more susceptible to functionalization, whereas the meta- and para-isomers display significantly greater chemical stability [23]. This diversity of hydrophilicity and polarity among the described borane clusters makes it possible to adjust the physicochemical properties of the compound, potentially influencing its behavior in biological systems. In Figure 2, the chemical structures of selected *closo*-boranes and *closo*-carboranes are presented, namely decahydro-*closo*-decaborate [B_10_H_10_]^2−^, dodecahydro-*closo*-dodecaborate [B_12_H_12_]^2−^, and carba-*closo*-dodecaborate [CB_11_H_12_]^−^, as well as the ortho-, meta-, and para-carboranes.

Boron clusters themselves generally do not display specific, high-affinity interactions with biological receptors or enzymes. Their interactions with biomolecules are typically weak or nonselective. For example [B_12_H_12_]^2−^ has been reported to exhibit no significant interaction with serum albumin [24]. Nevertheless, boron clusters are valuable components in drug design because chemical modifications enable their incorporation into pharmacophores capable of targeting specific biological receptors [25]. In addition, boron-cluster-containing compounds have shown potential as antimicrobial agents [26]. Although boron clusters have been studied in various biomedical contexts, their high boron content, stability, low toxicity and modifiability make them particularly suitable for boron neutron capture therapy (BNCT) [27], as described in more detail in Section 6.

## 3. Beneficial and Adverse Action of PAMAM and PLL Dendrimers

Dendrimers can be classified according to their chemical structure, function, and generation number. With respect to chemical structure, representative examples include poly(amidoamine) (PAMAM), poly(propyleneimine) (POPAM), and poly-L-lysine (PLL) dendrimers [28]. PAMAM dendrimers consist of a core and peripheral groups. Hydrophobic cores commonly used for PAMAM dendrimer synthesis include ethylenediamine, diaminododecane, diaminooxane, and diaminobutane [29,30]. The most frequently selected peripheral groups are amide, hydroxyl, or carboxyl functionalities [31].

### 3.1. Physicochemical and Biological Properties of PAMAM Dendrimers

In our previous studies [32,33,34], we demonstrated that second- and fourth-generation PAMAM-NH_2_ dendrimers with an ethylenediamine core act as effective stabilizers of gold nanoparticles, yielding colloids that remain stable for more than 18 months. These complexes outperform conventional iodine-based agents in terms of radiation absorption when used as contrast media in computed tomography (CT) imaging [35]. Due to their high atomic number, which improves image contrast, gold nanoparticles are good contrast agents. When combined with PAMAM dendrimers, they can further improve signal intensity and facilitate early cancer detection [36,37]. Another advantage of using PAMAM dendrimers in cancer therapy and other biomedical applications is their ability to enhance the bioavailability, pharmacokinetics, and pharmacodynamics of active compounds in the body [38]. Dendrimers facilitate selective and controlled delivery of drugs, vaccines, and genetic material [39,40,41]. Gene therapy remains a rapidly evolving field of medicine, particularly in the search for safe and effective carriers of genetic material that are non-toxic to patient cells and capable of delivering intact nucleic acids to target tissues. Due to the uncertain safety profile of biologically derived vectors, synthetic carriers such as PAMAM dendrimers are increasingly being considered as viable alternatives [42]. Nevertheless, due to various functional groups, PAMAM dendrimers themselves also exhibit high cytotoxicity [43], which depends on the size of the dendrimer, surface charge and generation number. Reports in the literature [44,45] indicate that lower-generation PAMAM dendrimers are less toxic than their higher-generation counterparts. Zeng et al. [46] demonstrated that PAMAM-NH_2_ dendrimers of generations higher than three significantly reduced cell viability and neuronal differentiation (human progenitor cells) at 5 μg/mL concentrations, whereas G0, G1, and G2 dendrimers exhibited no effect at this concentration. Moreover, the cytotoxicity indices of PAMAM-NH_2_ dendrimers at 10 μg/mL in neuronal cell cultures correlated with the zeta potentials of these nanostructures [46]. In our previous study [45], we demonstrated that the toxicity of PAMAM-NH_2_ dendrimers toward human umbilical vein endothelial cells (HUVECs) is generation-dependent—the higher the generation, the greater the cytotoxicity. At concentrations of 1.90, 0.45, and 0.35 μM/L for second-, fourth-, and seventh-generation PAMAM-NH_2_ dendrimers, respectively, a 25% reduction in HUVEC viability relative to control cell viability was observed, accompanied by a statistically significant increase in apoptotic cells and a decrease in the elasticity parameter encompassing both the cell membrane and the cortical cytoskeleton [45]. A reduction in cell elasticity within these ranges indicates actin fiber depolymerization processes, which may consequently lead to apoptosis. A similar relationship was observed for the immortalized endothelial EA.hy926 cell line exposed to fourth-generation PAMAM-NH_2_ dendrimers, where a concentration of 0.8 μM/L—resulting in a 25% reduction in cell viability—led to an increase in apoptotic cells, enhanced cellular elasticity, and elevated levels of reactive oxygen species [47]. These findings indicate that the toxicity of PAMAM-NH_2_ dendrimers is associated with the initiation of processes leading to apoptosis in endothelial cells.

### 3.2. PLL Dendrimers—Properties and Biological Activity

PLL dendrimers are based on lysine—an amino acid that enables the formation of multiple branching points [48]. Owing to their flexible structure and properties such as good biocompatibility, water solubility, and high resistance to proteolytic degradation, they may find applications in numerous areas of biomedicine.

Klajnert et al. [49] reported the synthesis of low-molecular-weight lysine-based peptide dendrimers that exhibited pronounced antimicrobial activity against Gram-positive bacteria (*Staphylococcus aureus*) and Gram-negative bacteria (*Escherichia coli*), as well as against fungal pathogens such as *Candida albicans*. It has been demonstrated that the steric arrangement and the nature of hydrophobic (aromatic) groups, as well as the types of cationic centers, constitute essential structural elements of dendrimers and influence both their antimicrobial activity and their toxicity [49]. Boyd et al. demonstrated the synthesis of PLL dendrimers radiolabeled with ^3^H [50]. Biodistribution studies conducted in rat models showed that the radioactivity of *L*-lysine-terminated dendrimers was broadly distributed across major organs, with no evident preference for components of the reticuloendothelial system [50]. Janiszewska et al. [51] reported the synthesis and characterization of amphiphilic poly-*L*-lysine dendrimers that can serve as *si*RNA carriers. These structures also inhibit the proliferation of two glioblastoma cell lines (C6 and U87) while exhibiting no toxicity toward normal neurons and glial cells, which coexist with glioblastoma cells within the affected brain tissue. Al-Jamal et al. [52] developed a synthetic approach for conjugating doxorubicin with sixth-generation cationic poly-*L*-lysine dendrimers (DOX-PLL). The therapeutic efficacy of these conjugates was demonstrated in a B16F10 melanoma mouse model, while their retention was confirmed in a Calu-6 lung cancer xenograft model in tumor-bearing mice. This study indicates that cationic PLL dendrimers—in addition to their systemic anti-angiogenic activity—are capable of complexing doxorubicin, thereby enhancing its accumulation and cytotoxicity in solid tumors in vivo [52]. Neelov et al. [53] performed computer simulations of third- and fifth-generation PLL dendrimers and examined the influence of these structures, as well as selected lysine-based hyperbranched polymers (HpbK), on the aggregation process of amyloid peptide in solution. Cell-viability assays and patch-clamp analyses demonstrated that third-generation PLL dendrimers can protect SH-SY5Y neuroblastoma cells against Aβ-induced cytotoxicity and K^+^-channel modulation [53].

### 3.3. DNA Damage Mechanism Induced by PAMAM and PLL Dendrimers

Reactive oxygen and nitrogen species (RO/NS) comprise a range of oxygen derivatives that play an essential role in normal physiological processes, such as cell signaling, gene expression regulation, proliferation, immune response, and maintenance of redox balance [54,55,56]. The primary source of ROS is oxygen metabolism within the mitochondrial matrix [54,55]. Under certain conditions, their concentration may increase to non-physiological levels, threatening proper cellular function. Excessive ROS leads to DNA damage, referred to as “oxidative stress,” which can compromise the integrity of both nuclear and mitochondrial DNA (*mt*DNA) [57,58]. Oxidative stress results from an imbalance between ROS production and antioxidant defense mechanisms [59,60,61]. Reactive oxygen species include: the superoxide anion radical ($O_{2}^{•-}$), the hydroxyl radical (^•^OH), singlet oxygen (^1^O_2_), ozone (O_3_), hydrogen peroxide ($H_{2}O_{2}$), and nitric oxide (NO) formed during *L*-arginine metabolism [62]. Among these, the hydroxyl radical (^•^OH) is considered one of the main contributors to oxidative DNA damage. Occurrence of the hydroxyl radical in the cell environment can lead to many undesirable reactions with macromolecules such as lipids, peptides or oligonucleotides, which results in their modification. Both $O_{2}^{•-}$ and ^•^OH can individually act on lipid membranes to promote formation of lipid radicals which, in the presence of oxygen, are capable of generating lipid peroxy radicals and they may involve membrane lipid peroxidation [63].

Dendrimers, particularly cationic PAMAM structures, generate ROS predominantly through their interactions with cellular and mitochondrial membranes [64]. The high density of surface-positive charges perturbs membrane integrity and disrupts intracellular homeostasis, ultimately leading to excessive ROS production and substantial cytotoxicity in healthy cells [45,47]. This ROS-mediated stress also underlies the ability of PAMAM dendrimers to induce DNA damage. Moreover, this effect intensifies with increasing dendrimer generation, and consequently with their larger size, higher degree of branching, and greater number of surface amine groups. The observed genotoxicity predominantly involves DNA strand breaks, frequently associated with mitochondrial dysfunction and the initiation of apoptosis [43]. At the same time, the ability of PAMAM dendrimers to bind DNA may confer a protective effect against other forms of degradation, rendering their overall impact complex and dependent on concentration, cell type, and surface chemical properties. PLL dendrimers were among the first cationic dendrimers used in complexes for drug release [52]. They can be successfully employed as siRNA carriers [51].

In the study by Naha et al. [65], increased intracellular production of reactive oxygen species (ROS), genotoxicity, and apoptosis were observed following the in vitro exposure of PLHC-1 fish hepatocellular carcinoma cells to G4, G5, and G6 PAMAM dendrimers. ROS generation and the genotoxic response increased linearly with the number of amine groups but reached saturation at higher doses. This study concluded that the observed genotoxicity, associated with DNA damage, is linked to low-level, generation- and dose-dependent intracellular ROS production. At higher ROS levels, the enhanced DNA damage correlates with the occurrence of necrosis in PLHC-1 cells [65]. Choi et al. [66], using the comet assay, demonstrated in Jurkat T-leukemia cells that PAMAM dendrimers, at concentrations ranging from 10 to 50 µg/mL and following a 4 h incubation, induce dose-dependent DNA single-strand breakage. In this model, an increased formation of micronuclei was also observed, as assessed using the cytokinesis-block micronucleus assay. The mechanism of DNA damage induced by PAMAM dendrimers has been associated with membrane “hole formation”, as evidenced by the lactate dehydrogenase (LDH) release assay [66]. Zeng et al. [46] demonstrated that fourth-generation PAMAM-50% C12 dendrimers (containing 50% amino groups and 50% *N*-(2-hydroxydodecyl) substituents), at a concentration of 1 µg/mL, altered the expression levels of oxidative-stress-related genes such as *ROR1*, *CYP26A1*, and *TGFB1*, which are implicated in the induction of DNA damage in human neural progenitor cells (HNPCs).

### 3.4. Strategies to Mitigate the Cytotoxicity of PAMAM and PLL Dendrimers

To mitigate the cytotoxic effects of PAMAM dendrimers, various surface-modification strategies are employed, such as polyethylene glycol conjugation [67,68] or the introduction of other chemical groups, including carboxylic acid or hydroxyl functionalities [69]. The highly branched architecture of dendrimers, characterized by numerous reactive amine groups [70], enables functionalization with diverse polymers or antibodies, thereby facilitating targeted therapeutic applications [71,72,73]. Kaul et al. [74] investigated PAMAM dendrimers and their pyrrolidone-modified hybrids as carriers for delivering 3,4-dihydroxybenzo-hydroxamic acid to the brain. This compound inhibits the enzyme ribonucleotide reductase, which can stop the synthesis of deoxyribonucleotides, but on its own it is unable to cross the blood–brain barrier. Based on the obtained results [74] the use of dendrimers was found to enhance the uptake of the acid by glioblastoma cells, with the most pronounced effect observed for pyrrolidone-PAMAM hybrid dendrimers. Moreover, hybrid dendrimers containing carboxyl groups exhibited superior biocompatibility compared with PAMAM-NH_2_ dendrimers [75].

Various strategies for modifying PLL dendrimers have also been reported in the literature, aiming to reduce their toxicity and to enhance their suitability for drug or gene delivery [76]. Chemical surface modification and functionalization of these nanomaterials have been shown to improve their therapeutic and diagnostic performance. Examples of PLL dendrimer applications in drug delivery are discussed in Section 5.1, *PLL Dendrimers*.

**Figure 3:**
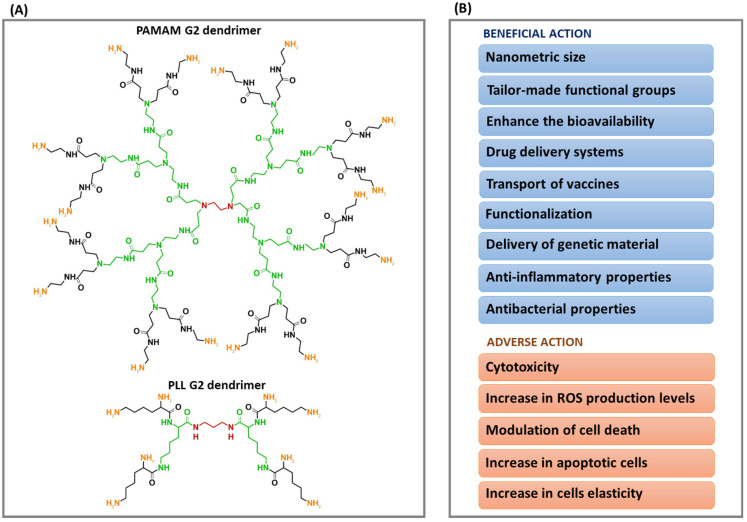
Chemical structure of 2nd-generation PAMAM and PLL dendrimers of (**A**), accompanied by a summary of their beneficial and adverse effects (**B**). The structure of 1st-generation dendrimers are highlighted in green. Orange markings indicate the functional groups of the dendrimers, while red denotes their core.

Figure 3 illustrates the chemical structure of PAMAM and PLL dendrimers of the second generation, along with the associated beneficial and adverse effects discussed in this section.

## 4. Internalization of Dendrimers

The biomedical application of PAMAM and PLL dendrimers continues to expand, and from this perspective, it is essential to elucidate the mechanisms governing their cellular uptake, intracellular transport, and the factors influencing these processes. In our previous study [45], we demonstrated that HUVECs incubated with PAMAM dendrimers contained single or multiple endocytic vesicles (lysosomes) of micro- and nanoscale dimensions, exhibiting regular multilayered structures. Such vesicles were not observed in the reference cells that were not exposed to PAMAM dendrimers. The multilayered structures inside the vesicles indicated the self-organization of PAMAM dendrimers [45]. The surface charge of dendrimers, which depends on both the core composition and terminal group identity, plays a decisive role in cellular uptake, biodistribution, and therapeutic performance. The size and morphology resulting from self-organization may affect: circulation time, accumulation in tumors via the Enhanced Permeability and Retention (EPR) effect and clearance by kidneys or the reticuloendothelial system. Well-controlled self-organization of PAMAM dendrimers enhance targeted delivery, while uncontrolled aggregation may lead to rapid clearance or off target accumulation.

**Figure 4:**
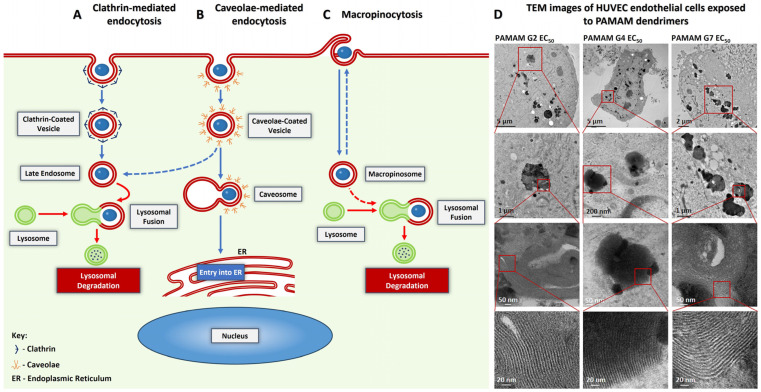
Pathways of PAMAM and PLL dendrimer uptake by cells (**A**–**C**) [77,78] and transmission electron microscope images (**D**) showing the self-organization of PAMAM G2, G4, and G7 dendrimers inside HUVECs at EC_50_ concentration (resulting in 50% of cellular viability). Figure (**D**) adapted with permission from Ref. [45]. 2022, Taylor & Francis. The consecutive bottom images in (**D**) present the enlarged areas from marked red squares. The multilayered structures observed inside the vesicles (in the lowest bottom images) indicate the self-organization of PAMAM dendrimers, and the measured distances between the bright and dark layers increase with dendrimer generation (amounting to 5.59 ± 0.41, 6.35 ± 0.44, and 7.59 ± 1.05 nm, respectively), which may be connected with the dendrimer diameter size [45].

Figure 4 presents the potential pathways of PAMAM and PLL dendrimers uptake by cells (Figure 4A–C) [77,78] together with transmission electron microscope images showing the self-organization of PAMAM dendrimers inside cells (Figure 4D) [45].

One possible pathway of PAMAM dendrimer transport is passive diffusion across the cell membrane [45,79,80]. When using an agent that increases the permeability of the lipid bilayer, nanopores are formed, which allow dendrimers to diffuse through the membrane [81]. The second pathway is endocytosis—an active transport mechanism involving the invagination of the cell membrane, which enables nanostructures to be enveloped into vesicles that are subsequently transported into the cytoplasm. In this active transport process, once internalized, intracellular trafficking routes determine the fate of the dendrimer cargo, directing it to specific organelles or facilitating its release into the cytosol [82,83]. Endocytosis of PAMAM and PLL dendrimers may be clathrin-dependent, where clathrins first bind to the cell membrane, and then an endocytic vesicle covered with clathrin and containing the dendrimer is formed [84,85]. In this mechanism, once internalized, clathrin-coated vesicles rapidly shed their coat and fuse with early endosomes; the subsequent stage involves the formation of late endosomes, which then merge with lysosomes to enable degradation (Figure 4A) [43,86]. Another endocytic mechanism involves caveolae, in which acaveolar vesicle containing the dendrimer is formed [84,87]. In this pathway, lysosomal degradation may be bypassed if the endosome is not directed toward the late endosomal route but instead trafficked to the endoplasmic reticulum through fusion with a caveosome (Figure 4B). In the context of dendrimer internalization, macropinocytosis should also be considered (Figure 4C) [84,88]. In this process, membrane ruffles envelop the dendrimer, forming an intracellular vacuole known as a macropinosome. Macropinosomes subsequently fuse with lysosomes to enable their degradation [89].

## 5. Dendrimers in Clinical Trials

Several approaches to the use of dendrimers as nanoscale drug or supplement carriers have been described in the literature. One of the more common strategies involves the incorporation (encapsulation) of hydrophobic molecules within their nonpolar core [90]. This enables poorly water-soluble compounds to become more compatible with aqueous environments, facilitating their administration and potentially improving their bioavailability [91]. Another strategy involves the covalent conjugation of the active substance to the dendrimer surface via functional groups located on the periphery [92,93]. This approach enhances stability and enables more precise control over both the mode and rate of drug release. Additionally, polymeric linkers can be incorporated into the structure to render the system responsive to specific stimuli. These stimuli may be external, such as changes in temperature or light irradiation, but can also originate from within the body—for example, local pH or redox conditions in the cellular microenvironment. Such a design enables precise control over the release profile of the active compound, substantially enhancing the therapeutic potential of dendrimers, particularly those of the PAMAM type, and positioning them as promising tools in nanomedicine.

PLL and PAMAM dendrimers are already incorporated into marketed pharmaceutical products, and formulations based on these structures are also undergoing clinical trials.

### 5.1. PLL Dendrimers

A representative example of a commercially available product is VivaGel^®^ (Starpharma Pty Ltd., Victoria, Australia), in which PLL dendrimers serve as the active pharmaceutical ingredient. The formulation is intended for the management of bacterial vaginosis and for the prevention of HIV and HSV infections [94,95]. Another commercially available product is VIRALEZE™ (Starpharma Pty Ltd.), which utilizes PLL dendrimers as an antiviral nasal spray intended for use in SARS-CoV-2 infection [96,97]. In this study, monomers derived from 1,3,5-benzenetricarboxylic acid were utilized, serving also as direct precursors for generating the charged dendrimer surface.

Another important application of PLL dendrimers is their use as contrast agents. A representative example is the commercially available contrast agent Gadomer-17 (Bayer Schering Pharma AG, Berlin, Germany), a PLL dendrimer conjugate containing 24 gadolinium ions within a single molecule. The molecule was designed to be large enough to remain in the blood vessel but small enough to be filtered by the kidneys. The contrast agent exhibits several potentially advantageous properties for Magnetic Resonance Angiography (MRA), most notably its high relaxivity [98].

Current clinical trials include tests for various conjugates of commonly used anticancer drugs and PLL dendrimers functionalized with polyethylene glycol (PEG-PLL) as drug delivery systems, which have been named Dendrimer Enhanced Product (DEP platform, Starpharma Pty Ltd.). One such example is the PEG-PLL dendrimer–docetaxel conjugate, marketed as DEP^®^ docetaxel (Starpharma Pty Ltd.), a result of which a prolonged plasma half-life, lower peak blood concentrations, and greater overall drug exposure were demonstrated in a Phase I clinical study compared with conventional docetaxel [99]. In a Phase II clinical trial, this formulation exhibited antitumor activity in patients with advanced metastatic cancers, including pancreatic cancer, gastric and esophageal cancers, non-small cell lung cancer (NSCLC), and cholangiocarcinoma [100]. Additionally, the product DEP^®^ cabazitaxel (Starpharma Pty Ltd.), a conjugate of a PEG-PLL dendrimer and the anticancer drug cabazitaxel, is currently in Phase II clinical trials for the treatment of solid tumors [101]. Another formulation currently being evaluated in Phase II clinical trials is DEP^®^ irinotecan (Starpharma Pty Ltd.), investigated for its anticancer activity in the treatment of colorectal, breast, ovarian, pancreatic, lung, and esophageal cancers [102]. Clinical studies have demonstrated sustained tumor reductions—lasting in some cases up to 72 weeks—as well as decreases in tumor marker levels for this product. Another anticancer candidate under investigation is *Re*-ImDendrim (French Association for the Advancement of Medical Research, Paris, France), a rhenium–nitroimidazole [^188^Re]-based ligand loaded onto a fifth-generation PLL dendrimer [103]. The formulation is intended for in situ treatment of unresectable liver cancer and is currently undergoing Phase I clinical evaluation to assess its safety and efficacy. A summary of products containing PLL dendrimers that are either commercially available or currently in clinical development is presented in Table 1.

### 5.2. PAMAM Dendrimers

PAMAM dendrimers are employed in the commercially available cardiac diagnostic test Stratus CS (Siemens Healthcare, Forchheim, Germany), a fluorometric immunoassay analyzer based on solid-phase radial immunoassay technology [77,104]. In this device, the dendrimer is immobilized on fiberglass paper onto which the first antibody is applied, followed by the whole-blood sample and the second antibody. Enzyme activity is initiated by the substrate wash solution, which simultaneously removes unbound labeled antibody. PAMAM dendrimers can also be used as gene-delivery vectors. Commercially available systems include SuperFect^®^ (Qiagen, Venlo, The Netherlands) and Priofect^®^ (Starpharma Pty Ltd., Victoria, Australia) [105]; however, these rely on high-generation dendrimers, which are associated with elevated costs and increased cytotoxicity. In the study by Liu et al. [106], preliminary non-commercial yet high-efficiency gene carriers were developed using second-generation PAMAM dendrimers cross-linked via disulfide bridges (referred to as a transfection reagent). The developed materials were capable of effectively condensing DNA into polyplexes of approximately 200 nm and degrading back to G2 dendrimers after cellular uptake. PAMAM dendrimers are also utilized in the PolyFect (Qiagen) transfection reagent, in which rapid and efficient DNA transfection of standard cell lines has been achieved, accompanied by high cell viability and low cytotoxicity [77,96]. Also noteworthy are drug-delivery systems based on PAMAM dendrimers that are currently undergoing clinical trials. One such product in Phase I clinical trials is OP-101 (Orpheris, Redwood City, CA, USA), which consists of *N*-acetylcysteine (NAC) linked via a disulfide bond to a PAMAM-OH G4 dendrimer [107]. This formulation was developed for the treatment of neuroinflammatory disorders, with its initial indication being childhood cerebral adrenoleukodystrophy. The disulfide linkage can be cleaved by glutathione, enabling intracellular release of NAC.

PAMAM dendrimers represent a promising solution to the current limitations of many positron emission tomography (PET) tracers used in neuroinflammation diagnostics. Studies on the tracer [^18^F]OP-801, which employs fluorescently labeled PAMAM-OH G4 dendrimers, have demonstrated that these compounds do not cross the intact blood–brain barrier; however, under inflammatory conditions they traverse the barrier, diffuse throughout the brain parenchyma, and are rapidly and highly specifically taken up by reactive microglia and macrophages via endocytosis [108]. They exhibit a high signal-to-background ratio and can be readily radiolabeled. [^18^F]OP-801 remains stable in human plasma, with the highest absorbed doses observed in the kidneys and bladder wall. A Phase I/II clinical trial is currently underway, involving first-in-human imaging both in healthy controls and in patients with amyotrophic lateral sclerosis. Products based on PAMAM dendrimers that are currently commercially available or in clinical development are summarized in Table 2.

Compared with other dendrimer classes, PLL and PAMAM structures exhibit the broadest spectrum of therapeutic applications, encompassing antibacterial, antiviral, and antioxidant activities, as well as diagnostic utility [38]. Despite the ongoing discussion in the literature regarding nanoparticle safety, the accumulated preclinical and clinical data on polymeric excipients, biomedical polymers, and polymer-based therapies indicate that the targeted development of dendrimer chemistry within selected application areas may lead to the creation of safe materials with high value for biomedical and pharmaceutical use.

## 6. Boron Neutron Capture Therapy (BNCT)

One of the main components of BNCT is the application of boron-containing compounds. In nature, boron mainly occurs in the form of salts of boric acid, most commonly borax. There are two stable isotopes of boron, B511 and B510, which have natural abundances of approximately 80% and 20%, respectively [109]. In the context of BNCT, the crucial isotope is ^10^B, due to its [109] high thermal neutron capture cross-section, reaching approximately 3840 barns at a neutron energy of 0.25 eV, in stark contrast to the markedly lower value of about 0.33 barns observed for H11. The compound serving as a boron carrier is required to fulfill several critical criteria. It must demonstrate sufficient aqueous solubility to permit safe and effective systemic loading, because it is administered intravenously [110]. Furthermore, high tumor selectivity is essential to achieve tumor-to-normal tissue and tumor-to-blood concentration ratios of no less than 3–4 [111]. The agent should also ensure adequate intratumoral accumulation of B510, typically within the range of approximately 20–35 µg/g [112]. Equally important is its capacity for sustained cellular retention, enabling the compound to remain within malignant cells for an appropriate duration and preventing premature clearance prior to neutron irradiation [113].

Another essential component of BNCT is the neutron source. In the past, nuclear reactors served as the primary neutron generators, providing high fluxes of thermal and epithermal neutrons; however, their clinical use was constrained by substantial infrastructural demands and stringent regulatory limitations [114]. In recent years, accelerator-based neutron sources (ABNSs) have emerged as a viable alternative, enabling installation within hospital settings and facilitating more streamlined regulatory approval processes [115]. Their development is now a key factor enabling wider implementation of BNCT in clinical practice.

**Figure 5:**
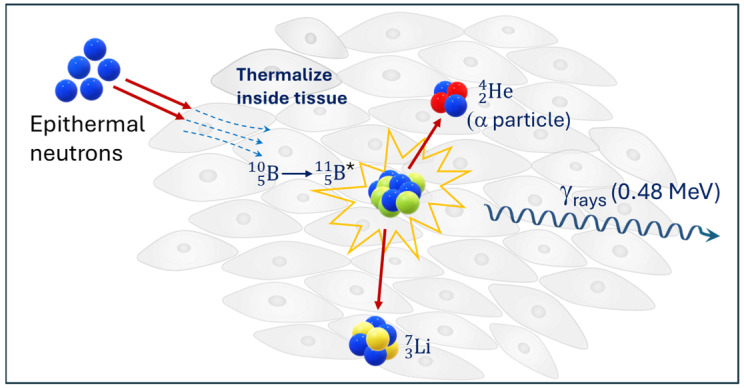
The fundamental nuclear reaction between boron, B510, and low-energy thermal neutrons [91,98]. * indicates the excited state of boron B511.

Figure 5 presents nuclear reaction between B510 and low-energy thermal neutrons. Following administration of the boron carrier, the tumor region is irradiated with a beam of epithermal neutron energy (E) from 0.5 eV to 10 keV, which is subsequently moderated within the tissue to thermal energies (E < 0.5 eV). These thermal neutrons are captured by B510, initiating the nuclear reaction B510, ^1^*n*, α (He24), Li37 and resulting in the emission of high-energy particles—the Li37 nucleus and α particle—accompanied by a minor release of γ radiation (0.48 MeV) [116]. The reaction products exhibit a high Linear Energy Transfer (high-LET) of 150–200 keV/μm and a short penetration range of approximately 5–9 μm [117]. This distance corresponds to the diameter of a single cell, ensuring that cytotoxic effects are confined almost exclusively to malignant cells while sparing the surrounding healthy tissue.

The first generation of boron-containing compounds, selected primarily due to their broad availability, included boric acid (H_3_BO_3_) and borax (Na_2_B_4_O_7_·10H_2_O). However, clinical studies demonstrated their insufficient tumor accumulation and considerable toxicity, which resulted in substantial damage to healthy tissues [118,119].

The second generation, still used in contemporary BNCT research and practice, is characterized by markedly lower toxicity and comprises sodium mercaptoundecahydro-*closo*-dodecaborate (Na_2_B_12_H_11_SH, BSH) and (*L*)-4-dihydroxy-borylphenylalanine (*L*-BPA) [120]. BSH contains as many as 12 boron atoms per molecule, yet its tumor selectivity is limited, and its intratumoral accumulation remains low due to passive diffusion. In contrast, *L*-BPA exhibits significantly higher tumor selectivity and markedly greater accumulation within cancer cells. This enhanced uptake is largely attributable to its structural similarity to amino acids, enabling transport predominantly via the *L*-type amino acid transporter (LAT). Nevertheless, *L*-BPA contains only a single boron atom per molecule and displays poor water solubility [118,119].

The third generation comprises newly developed compounds designed to enhance tumor selectivity by incorporating targeting moieties such as peptides, proteins, antibodies, nucleosides, sugars, porphyrins, liposomes, nanoparticles, or dendrimers, while simultaneously retaining a high density of boron-containing groups [118,119]. In parallel, additional functional groups are being integrated to enable imaging capabilities, thereby facilitating more effective monitoring of carrier accumulation within tissues.

## 7. PAMAM and PLL Dendrimers Functionalized with Boron Clusters

In this section, we discuss the current state of knowledge on borane cages in the context of their application with nanostructures—specifically PAMAM and PLL dendrimers—for BNCT. The idea behind using borane cages with PAMAM dendrimers was to conjugate them with monoclonal antibodies. Monoclonal antibodies are widely used in the treatment of various cancers. They are characterized by highly specific targeting of defined biological markers, enabling the recognition of particular proteins expressed on the membranes of cancer cells.

The first experimental approach [121,122] involved the functionalization of poly-*DL*-lysine with isocyanate derivatives of *closo*-boranes, which were then conjugated with monoclonal antibodies. These studies were the result of earlier experiments in which 1-isocyanate-closo-dodecaborane and 2-trimethylamino-7(8)-isocyanate-*closo*-decaborane were directly attached to anti-thymocyte globulin (ATG). To prevent loss of immunoreactivity, a linker strategy based on poly-*DL*-lysine was introduced. The resulting conjugates contained more than 1000 boron atoms per macromolecule and retained 58% and 40% of their immunoreactivity for IB 16-6 and 17-1A, respectively. These syntheses were successful due to the high reactivity of isocyanate groups towards amine groups present in anti-bodies. Unfortunately, the resulting conjugates lost their immunoreactivity. The same strategy was later applied to human anti-TSH and nonspecific polyclonal IgG antibodies, which were perio-donated before reactions. Formed conjugates preserved their in vitro immunoreactivity and contained approximately 6000 boron atoms per macromolecule [121,122]. Thus, it can be concluded that this strategy minimized the loss of monoclonal antibody immunoreactivity in the presence of borane clusters, making them promising candidates as delivery systems for BNCT. The drawback of such systems is the high polydispersity of the polymer molecular mass and thus the heterogeneity of the boron atoms present.

Barth et al. [123] proposed PAMAM G2 and G4 dendrimers functionalized with a decaborane cluster. Due to their hyperbranched structure, PAMAM dendrimers were a preferred solution, exhibiting lower molar-mass dispersity. The boronated PAMAM dendrimers were derivatized using m-maleimidobenzoyl-*N*-hydroxysulfosuccinimide ester (sulfo-MBS) and subsequently conjugated to the monoclonal antibody IB 16-6 (targeting the murine B16 melanoma cell line), which had been derivatized with *N*-succinimidyl 3-(2-pyridyldithio)propionate (SPDP) [123]. The antibody was linked to the outer shell of the dendrimer through a modified site, which prevented random attachment and preserved its localization properties in in vitro studies. In in vivo studies conducted in both healthy and tumor-bearing mice—C57Bl/6 models with subcutaneous B16 melanoma implants—accumulation of the boronated PAMAM-IB16-6 dendrimers was confirmed in the liver and spleen, along with a lack of tumor specificity. These findings indicate that further modifications of the boronated dendrimers are required to reduce this nonspecific accumulation [123].

Wu et al. [124] investigated highly boron-loaded PAMAM G5 dendrimers. In this study they examined the use of the chimeric monoclonal antibody cetuximab, directed against the epidermal growth factor receptor (EGFR), as a boron carrier for NCT of brain tumors (F98WT and F98EGFR) in the Fischer rat model. The EGFR gene is frequently amplified in human gliomas, and tumor cells may express up to 100-fold higher levels of the receptor compared with normal cells. It can be targeted therapeutically by using EGF bioconjugates to deliver boron atoms to cancer cells. The highly boron-loaded PAMAM G5 dendrimer was conjugated to oligosaccharide residues located distal to the cetuximab antigen-binding sites, using the heterobifunctional reagents *N*-succinimidyl 3-(2-pyridyldithio)propionate (SPDP) and *N*-(κ-maleimidoundecanoyl)hydrazide (KMUH) [124]. Based on the relative ratios of boron to protein concentrations, it was found that there are approximately 1100 boron atoms per cetuximab molecule. The results confirmed highly specific EGFR targeting—92.3 (23.3) µg B/g of tumor tissue was detected in F98EGFR gliomas, in contrast to the low levels observed in normal brain tissue [124]. In turn, Capala et al. [125] obtained stable bioconjugates containing PAMAM G4 dendrimers with 960 boron atoms per molecule. The PAMAM G4 dendrimers were functionalized at their terminal amine groups with a polyhedral isocyanato-borane and subsequently equipped with a thiol group to enable reaction with the maleimide groups of epidermal growth factor (EGF) derivatives [125]. Moreover, electron microscopy demonstrated that the boronated PAMAM G4–EGF conjugate initially bound to the surface membrane of U-343MG human malignant glioma cells and subsequently underwent endocytosis, which ultimately led to the accumulation of boron in lysosomes. In a subsequent manuscript, Barth et al. [123] compared the efficacy of boronated EGF, also when used in combination with boronophenylalanine (a carrier targeting the EGFR). The boronated PAMAM G4 dendrimer was chemically conjugated to EGF using heterobifunctional reagents. One of the stages of the study [123] involved demonstrating that an antibody molecule must deliver approximately 1000 boron atoms in order to achieve the critical local boron concentration, taking into account the receptor site density on tumor cells and the affinity constant. The biodistribution of borated PAMAM G4-EGF was performed on a Fischer rat model for two rat glioblastoma cell lines, F98WT and F98EGFR. After 6 h, equivalent amounts of the bioconjugate were detected in both tumor types. At 24 h after stereotactic injection, boron concentrations in F98WT and F98EGFR tumor cells were 9.2 and 21.1 μg/g of tumor tissue, respectively, while no boron was detected in the liver, spleen, kidneys, brain, or blood. In the Fischer rat model (F98EGFR tumors), the mean survival time following BNCT was longer for the bioconjugate compared with boronophenylalanine (45 vs. 39 days) [123].

Yang et al. [126] evaluated convection-enhanced delivery (CED) to increase the uptake of a highly boron-loaded PAMAM G4–EGF bioconjugate by a tumor. The high molecular weight of the dendrimer–EGF bioconjugate is a limiting factor for its penetration into the blood–brain barrier. CED is a delivery method in which a pressure gradient is applied to establish bulk flow through the brain interstitium during infusion. This technique bypasses the BBB, enabling direct introduction of therapeutic agents into the extravascular space of the central nervous system. In a Fischer rat model bearing EGFR-expressing gliomas (F98EGFR), it was demonstrated [126] that delivery via CED was superior to intravenous administration, resulting in a greater intratumoral volume of distribution.

Backer et al. [127] presented a boron-functionalized PAMAM G5 dendrimer designed to generate a macromolecule containing 1050 to 1100 boron atoms per dendrimer, which was subsequently conjugated to the thiol groups of VEGF. To enable fluorescence imaging, the bioconjugate was labeled with the near-infrared Cy5 dye (VEGF-BD/Cy5 bioconjugate). Fluorescence imaging confirmed the selective accumulation of VEGF-BD/Cy5 (but not BD/Cy5) in the 4T1 murine breast tumor model, particularly at the tumor margins where angiogenesis was most active [127].

Shukla et al. [128] designed folate receptor (FR)-targeted, boronated PAMAM G3 dendrimers and used PEGylation to tune biodistribution, with the goal of delivering therapeutic boron selectively for BNCT while limiting reticuloendothelial uptake. Chemically, 12–15 decaborate clusters were grafted to G3 PAMAM and then modified with PEG chains; 1–1.5 PEG2000 units minimized hepatic uptake in mice, and an FA terminated construct showed FR dependent uptake in FR positive KB cells in vitro. In FR-positive 24JK FBP tumor-bearing mice, the FA PEG dendrimer achieved selective tumor uptake (~6% ID·g^−1^) but also high liver (~38.8% ID·g^−1^) and kidney (~62.8% ID·g^−1^) accumulation, indicating that further surface optimization is needed to balance targeting with off target retention [128]. Sun et al. [129] explored a receptor-targeted nanocarrier—a CD133-directed PAMAM dendrimer encapsulating BSH—to improve boron delivery to glioma stem-like cells as a strategy to enhance the effectiveness of BNCT. Across in vitro assays and orthotopic mouse models (SU2), the conjugate demonstrated preferential uptake in CD133-positive cells/tumors and, when paired with BNCT (often alongside systemic BSH), yielded higher tumor boron and longer survival versus the benchmark boron agent alone [129].

Yang et al. [130] evaluated a boronated PAMAM dendrimer–monoclonal antibody bioconjugate (BD–L8A4), targeted to EGFRvIII in a syngeneic F98 npEGFRvIII rat glioma model. It was demonstrated that convection-enhanced delivery (CED) provides significantly greater intratumoral retention than direct intratumoral injection (60.1% vs. 43.7% injected dose per gram of tissue at 24 h), with minimal uptake in normal tissues [130]. Table 3 provides a comparative overview of key boronated PAMAM dendrimer studies on the functionalization used and major findings from in vitro/in vivo experiments.

A promising strategy also involved the functionalization of poly-*L*-lysine dendrimers with carborane clusters [131]. Such lysine-based dendritic architectures offered the advantage of enabling the multivalent attachment of carboranyl amino acids through a single acylation step. Nevertheless, this concept was not further pursued in subsequent studies. To date, the majority of boron-functionalized dendritic structures evaluated in biological studies have been based on high-generation PAMAM dendrimers, typically generations four or five. Consequently, dendrimers capable of incorporating a substantial boron payload while simultaneously accommodating targeting moieties appear to represent the most practical and effective design strategy for the biological application of boronated dendrimers. Although the modification of PAMAM and PLL dendrimers with boron clusters has been discussed in the literature for many years, these systems may now warrant renewed consideration as boron nanocarriers for BNCT, particularly in light of the increasing clinical accessibility of this therapeutic modality. Nevertheless, practical application of boron-functionalized dendritic structures still requires further development and optimization.

## 8. Conclusions

Despite the intense interest in PAMAM- and PLL-type dendrimers, only a limited number of formulations incorporating these structures have progressed to clinical studies, and consequently, few are commercially available. The implementation of nanotechnology-based products in clinical practice depends on multiple factors, including the quality of design in the research and development stage, the use of appropriate preclinical models, the establishment of consistent experimental protocols, and the availability of adequate funding.

Another strategy for the use of PAMAM dendrimers of the second, third, fourth, and fifth generations involves their functionalization with borane clusters, which may serve as an attractive tool for cancer treatment using BNCT. The advantage of employing PAMAM dendrimers, compared with poly-*DL*-lysine, stems primarily from their lower molar-mass polydispersity and reduced heterogeneity in boron atom content. The search for new B510 carriers—particularly those capable of delivering a high number of boron atoms—aims to enhance the therapeutic efficacy of BNCT. The development of innovative, multifunctional therapeutic agents opens the possibility of implementing treatment strategies monitored in real time under imaging guidance. Recent data confirm that achieving high therapeutic precision, ensuring substantial local tumor control while maintaining acceptable toxicity, constitutes the radiobiological foundation for translating BNCT into clinical practice.

The obtained conclusions are outlined in the following points:

- Despite the broad interest in PAMAM and PLL dendrimers, particularly as drug-delivery vehicles, only a limited number of products based on these nanostructures are commercially available, largely due to high production costs and the need for optimized synthesis conditions.
- The mechanisms governing the internalization of PAMAM and PLL dendrimers into cells are well characterized, which may further support their potential biomedical applications.
- Due to limited access to neutron sources (including the need to construct accelerator-based facilities), BNCT is not yet a widely used therapeutic modality for oncology patients. Another major challenge for BNCT is the development of drug carriers that can be labeled and that selectively accumulate in target cells (tumors).
- To date, *L*-BPA and BSH remain the only boron-delivery agents used in BNCT that have demonstrated sufficiently promising in vivo results in terms of biodistribution, toxicology, and therapeutic efficacy.
- Previous studies conducted on animal models have shown that PAMAM dendrimers functionalized with boron clusters may serve as attractive tools for BNCT. Owing to their highly branched architecture, they can accumulate a large number of boron atoms within a single macromolecule (approximately 1000 boron atoms), which directly contributes to BNCT effectiveness.

## Figures and Tables

**Table 1:** Overview of commercially available PLL-based dendrimer products or those currently undergoing clinical trials.

Product Name	Applicability	Source
VivaGel^®^	management of bacterial vaginosis and prevention of HIV and HSV infections	[94,95]
VIRALEZE™	antiviral nasal spray intended for use in SARS-CoV-2	[96,97]
Gadomer-17	MRA contrast agent	[98]
DEP^®^ docetaxel	Phase II clinical trials—pancreatic, gastric, esophageal, non-small cell lung cancer, and cholangiocarcinoma	[100]
DEP^®^ cabazitaxel	Phase II clinical trials—treatment of solid tumors	[101]
DEP^®^ irinotecan	Phase II clinical trials—colorectal, breast, ovarian, pancreatic, lung, and esophageal cancers	[102]
*Re*-ImDendrim	Phase I clinical trials—unresectable liver cancer	[103]

**Table 2:** Overview of commercially available PAMAM-based dendrimer products or those currently undergoing clinical trials.

Product Name	Applicability	Source
Stratus CS	cardiac diagnostic test	[77,104]
SuperFect^®^, Priofect^®^	gene-delivery vectors	[105]
PolyFect	DNA transfection agent	[77,96]
OP-101	Phase I clinical trials—drug delivery system of NAC	[107]
[^18^F]OP-801	PET contrast agent	[108]

**Table 3:** Boronated PAMAM dendrimers as delivery systems for boron neutron capture therapy (BNCT).

PAMAM Generation	Functionalization; Boron Loading	Cell Culture/ Animal Model	Major Findings	Source
G2, G4	sulfo-MBS; mAb: SPDP; ~1690–8150 B atoms per antibody	B16 melanoma in C57BL/6 mice	high RES uptake; poor tumor localization (≤0.6% ID g^−1^)	[123]
G5	Fc-region oligosaccharide chemistry (SPDP, KMUH); Cetuximab; ≈ 1100 B atoms per cetuximab molecule	F98WT and F98 EGFR glioma cells; Fischer rats (intracerebral implants)	high specific EGFR targeting; 92 µg B g^−1^ (EGFR^+^); low normal brain tissue	[124]
G4	sMBS (maleimide) on EGF; thiol–maleimide coupling; 960 B atoms per EGF	malignant glioma U-343MG (human) and C6 EGFR (rat) cells	EGFR binding retained; rapid uptake; lysosomal localization	[125]
G4	EGF; 1000 B atoms per EGF	F98WT and F98 EGFR glioma cells; Fischer rats (intracerebral implants)	F98EGFR rat model—BNCT resulted in a longer survival time compared with BPA	[123]
G4	SPDP/DTT thiol generation; sMBS on EGF	F98 EGFR/F98 WT rat gliomas	CED boosts distribution ~6–7×; 24 h 47.4% ID g^−1^	[126]
G5	VEGF-Cy5; 1050 -1100 B atoms	BALB/c mice with 4T1 breast tumors	selective accumulation has been confirmed; perivascular accumulation	[127]
G3	optimal PEGylation ~1–1.5 PEG2000; FA at distal end	C57BL/6 mice bearing 24JK FBP sarcomas	selective tumor accumulation (~6% ID g^−1^) and high liver/kidney uptake	[128]
G5	anti-CD133 mAb; 1:1 antibody:dendrimer ratio	CD133^+^/CD133^−^ SU2 and U87s GSCs; BALB/c nude mice (orthotopic SU2)	selective CD133^+^ uptake; extension the survival time of tumor mice	[129]
G4/G5	EGF (anti-EGFRvIII mAb L8A4)	EGFRvIII-positive rat glioma; syngeneic Fischer rats	therapeutic efficacy CED, 24 h: 60.1% ID g^−1^ (EGFRvIII) vs. 14.6% (WT); low normal-tissue boron (<0.5 µg/g)	[130]

Abbreviations: PAMAM, poly(amidoamine); BPA, p-boronophenylalanine; EGF, epidermal growth factor; EGFR, epidermal growth factor receptor; EGFRvIII, mutant epidermal growth factor receptor; mAb, monoclonal antibody; SPDP, *N*-succinimidyl 3-(2-pyridyldithio)propionate; sMBS, m-maleimidobenzoyl-*N*-hydroxysulfosuccinimide; KMUH, *N*-(κ-maleimidoundecanoic acid) hydrazide; BSH, sodium mercaptoundecahydrododecaborate; FR, folate receptor; CED, convection-enhanced delivery; RES, reticuloendothelial system. Units: µg B g^−1^; % ID g^−1^ (percent of injected dose per gram of tissue).

## Data Availability

No new data were crated or analyzed in this study.

## Abbreviations

The following abbreviations are used in this manuscript:

ADME	absorption, distribution, metabolism, and excretion
PAMAM	poly(amidoamine)
BPA	p-boronophenylalanine
NAC	*N*-acetylcysteine
ID	injected dose
PLL	poly-*L*-lysine
POPAM	poly(propyleneimine)
EGF	epidermal growth factor
EGFR	epidermal growth factor receptor
EGFRvIII	mutant epidermal growth factor receptor
mAb	monoclonal antibody
SPDP	*N*-succinimidyl 3-(2-pyridyldithio)propionate
sMBS	m-maleimidobenzoyl-*N*-hydroxysulfosuccinimide
KMUH	*N*-(κ-maleimidoundecanoic acid) hydrazide
BSH	sodium mercaptoundecahydrododecaborate
FR	folate receptor
CED	convection-enhanced delivery
RES	reticuloendothelial system
BNCT	boron neutron capture therapy
TEM	Transmission Electron Microscopy
DEP	Dendrimer Enhanced Product
MRA	Magnetic Resonance Angiography
ROS	reactive oxygen species
ATG	anti-thymocyte globulin
PEG	polyethylene glycol
ER	endoplasmic reticulum
EPR	Enhanced Permeability and Retention
